# A case report: *Actinobaculum suis* infection associated with formation of pyogranuloma, epididymitis and azoospermia in a boar

**DOI:** 10.1186/s12917-020-02680-1

**Published:** 2021-01-06

**Authors:** Mirjam Arnold, Olivia Richard, Corinne Gurtner, Heiko Nathues, Alexander Grahofer

**Affiliations:** 1 Department of Clinical Veterinary Medicine, Clinic for Swine, Vetsuisse Faculty, University of Bern, CH Bern, Switzerland; 2 Department of Infectious Diseases and Pathobiology, Vetsuisse Faculty, Institute of Animal Pathology, University of Bern, CH Bern, Switzerland

**Keywords:** Testis, Andrology, Return to oestrus, Pig, Swine, Reproduction

## Abstract

**Background:**

*Actinobaculum suis* is a bacterium known to cause infections of the urogenital tract of sows. Infection can occur through close contact to boars, who frequently carry the pathogen in their preputial diverticulum but do not become clinically diseased themselves. In the current case, *Actinobaculum suis* was isolated from pyogranuloma of inflamed epididymis in a boar with poor fertility.

**Case presentation:**

Increased return to oestrus rate, which worsened after the purchase of a new boar, was reported in an organic farm in Switzerland. During herd examination, azoospermia of the boar was diagnosed, and slaughter, followed by examination of its urogenital tract, was carried out. Pathologically, pyogranuloma formation and epididymitis were diagnosed. Bacteriology of the pyogranulomas showed growth of *Actinobaculum suis *and mixed flora. After the boar was replaced, the return to oestrus rate improved tremendously.

**Conclusion:**

A close relative of *Actinobaculum suis, *namely *Actinotignum schaalii,* has already been associated with epididymitis in humans. Considering the present case and the parallels in human medicine, *Actinobaculum suis *should be included in the list of differentials of boars with poor fertility.

## Background

*Actinobaculum suis* [1], formerly known as *Corynebacterium suis, Eubacterium suis* [2] and *Actinomyces suis comb. Nov.* [3] is an anaerobic bacterium which can cause multiple infections of the urogenital tract in sows. The prevalence of *Actinobaculum suis* in pigs varies between countries and farms. In Switzerland, it was detected in 4% of the urinary bladders of slaughtered sows [4]. It is also described that the presence of the pathogen is not always associated with a urinary tract infection. One study even described, that sows positive for *Actinobaculum suis*, showed urinary tract infections less often compared to sows negative for the bacterium [5]. Noteworthy, severe urethritis and pyelitis/pyelonephritis are only expected in mixed infections with *Actinobaculum suis* and other bacteria [6]. In order to prevent disease transmission from boars to sows, artificial insemination is widely practiced in pig industry [7]. This is also recommended to reduce the spread of *Actinobaculum suis*, since its frequent presence in the preputial diverticulum of the boar was demonstrated in several studies [8–12]. In all of these studies, the percentage of positive preputial swabs was higher than 60% without boars reported to be diseased. Attempts to eliminate the pathogen from the diverticulum of carrier boars have been unsuccessful. Neither local nor systemic antibiotic administration or hygiene measures lead to long term persistent freedom of *Actinobaculum suis* in the diverticulum [13].

This is the first report describing a case of pyogranuloma and epididymitis in a boar with reduced fertility associated to *Actinobaculum suis.*

## Case presentation

The case occurred 2019 in an organic farrow to weaning farm in Switzerland, which reported reproductive problems. The farm consisted of 80 sows and one boar. The latter one was replaced in May 2019. Due to an increase of the return to oestrus rate to approximately 50% in August 2019, the Clinic for Swine at the Vetsuisse Faculty, University of Bern, was contacted to perform a herd examination. Return to oestrus rate was reported to be equally in gilts and sows, and regular (i.e. between day 18–24 and 36–48 after insemination) in about 80% of all cases. At this time point, no electronic data of the reproductive performance was available, therefore no further analysis of the data was conducted. During herd examination, all sows revealed a good general health and appropriate body condition. For further diagnostics, an andrological investigation including an ultrasound examination of the boar’s testicles was conducted and no abnormalities were found. Due to the absence of a sow in heat, no further examinations of the sexual behaviour and semen quality could be carried out. Semen for artificial insemination, which was used in about 70% of performed inseminations, was stored in a storage box at 17 °C. The management of insemination was examined and need for optimization in heat control and time of insemination were determined. Sow management, genetics and housing were identified as unlikely causes of the problem.

For further evaluation of the reproductive performance, the farmer provided current data. The analysis of the performance characteristics revealed an increased return to oestrus rate of 35.3% within the last nine months (Table 1).

**Table 1:** Performance characteristics of the sows on farm of the last 9 months (January- September 2019) and corresponding reference values

Performance characteristics	Status quoOctober 2019	Referencevalues [14]
Piglets born alive/ litter	13.8	12–14.0
Piglets born dead/ litter	1.8	≤ 0.5
Piglets weaned/ litter	11.1	11.0
Litter/ sow/ year	2.1	2.1^a^
Return to oestrus rate (%)	35.3	≤ 12.0
Regular return (%)^b^	28.7	
Irregular return (%)^c^	6.6	

^a^ calculated with 115 days pregnancy, suckling period of 56 days and 4 days return to oestrus

^b^ Sows returning to oestrus 18–24 and 36–48 days after insemination

^c^ All other sows returning to oestrus without regular return

The evaluation of the three months before the new boar was introduced (February to May 2019), showed a return to oestrus rate of 17.8%. After the introduction of the boar, the return to oestrus rate tremendously increased to 59.4%. Overall, 75.0% amongst naturally mated sows returned to oestrus. The remaining 25.0% of naturally mated sows remained unclear, as no pregnancy diagnostic was conducted on the farm. Overall, the findings of the analysis of the performance characteristics (Table 1) indicated the new boar to play a major role in the increased return to oestrus rate on farm. To assess the sexual behaviour and the sperm quality an investigation of the boar was conducted in October 2019. No abnormalities were detected in the mating behaviour. The ejaculate was collected, and a semen analysis was performed immediately (Table 2).

**Table 2:** Results of the semen analysis of the boar, which was suspected to influence the increased return to oestrus rate among sows in an organic pig farm

Characteristics	Semen analysis of the boar	Reference values [15]
Colour	Brown, reddish	White
Consistency	Watery	Watery – milky
pH	8.5	6.6–7.7
Additives	Blood +(+) Bilirubin ++	None
Sperm concentration	None	0.3 × 10^6^/µl

**Fig. 1:**
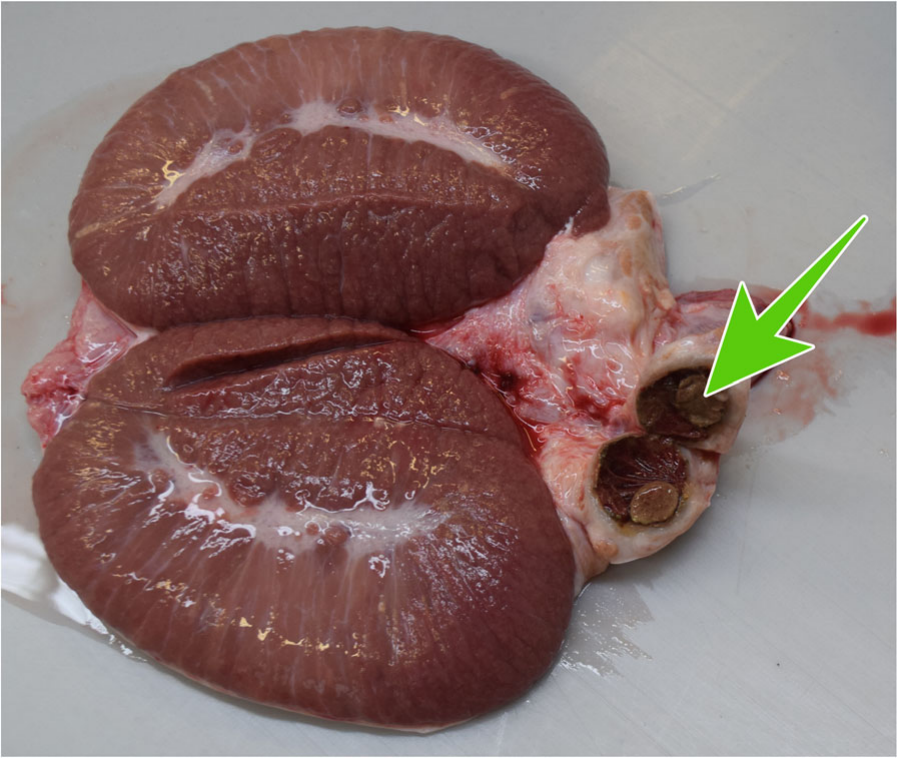
Testicle and epididymis of the slaughtered boar with cystic cavity in the head of the epididymis (green arrow)

**Fig. 2:**
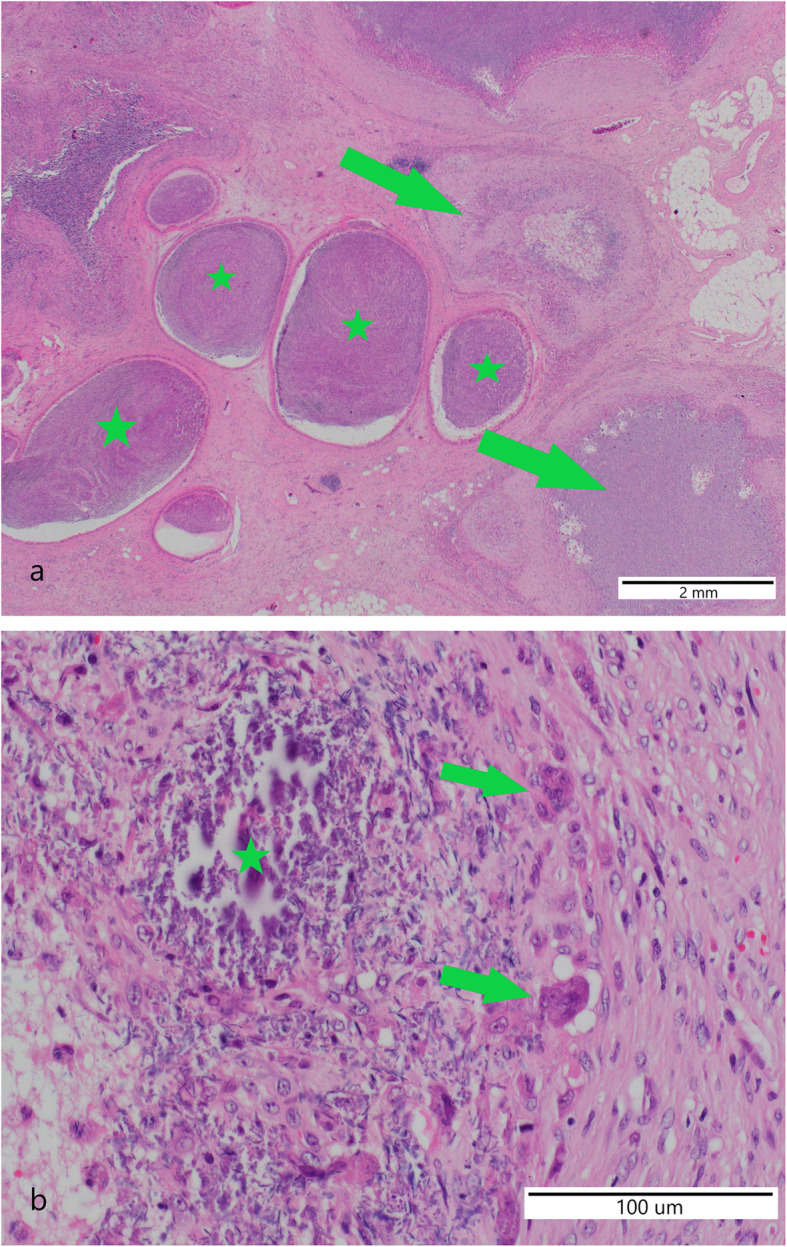
Histology (H&E stain) of the epididymis of the slaughtered boar. **a** Pyogranuloma (green arrows) and unaffected Ductus epididymidis (green stars). **b** Close up of a pyogranuloma: Mineralized centre (green star) surrounded by many multinucleated giant cells (green arrows) and proliferating fibroblasts (foreign body reaction)

Based on the results of the analysis of the performance characteristics and the findings of the andrological examination, it was concluded that the boar caused the reproductive failure in the naturally mated sows. Therefore, slaughter of the boar was recommended and performed. The urogenital tract was forwarded to the Institute of Animal Pathology at the University of Bern. Bladder, urethra, genital glands and penis were without gross changes. Bilaterally the epididymis was slightly enlarged with a diameter of up to 4 cm. The epididymal tissue was replaced by multiple cavities filled with beige, friable material. In the head of one epididymis, a round cystic cavity of 1.5 cm in diameter was present (Fig. 1). Bilaterally, approximately 5% of the testicular parenchyma was multifocally beige in colour and of slightly increased consistency. In histology, 60% of the normal architecture of the epididymis was destroyed by a chronic pyogranulomatous inflammatory reaction (Fig. 2).

The pyogranuloma originated in the *Vasa efferentis* and the *Ductus epididymidis*. They consisted of a necrotic centre with few spermatozoa, surrounded by few multinucleated giant cells, degenerated neutrophilic granulocytes and a seam of macrophages with lymphocytes and plasma cells, encapsulated by a thick layer of connective tissue. Multifocally in the inactive *Vasa efferentis*, intact spermatozoa were seen. Approximately 50% of the tubules of the testes were multifocally atrophied. The remaining tubules were filled with sperm in different stages and surrounded by active Leydig cells. The urethra, the *Glandula vesicularis* and the *Glandula bulbourethralis* were histologically without special findings. A Ziehl- Neelsen stain for acid-fast bacteria was negative. No fungal or parasitic organisms were detected in a PAS respectively Gram stain. The pathological changes were compatible with a bilateral, severe, multifocal to coalescing, chronic, granulomatous and necrotizing epididymitis with giant cells and multifocal atrophy of the tubules in the testicles.

A bacteriological examination of the epididymis was carried out at the Institute of Veterinary Bacteriology, Vetsuisse Faculty, University of Bern. In the bacteriological examination of the epididymis, a moderate amount of *Actinobaculum suis* and a low amount of mixed flora were isolated. Detection of *Brucella suis* via stamp staining and isolator Microbial Tubes enrichment procedure was negative.

### Follow-up

A breeding examination of the new boar, purchased in October 2019, revealed no abnormalities in behaviour or semen quality. Furthermore, a clinical examination of eight sows was performed, showing that sows were in good general health condition. In addition, spontaneous urine was examined macroscopically and by using a urine test strip (HS Urispec Plus 9 + Leuko®), revealing no abnormalities. Five months after the initial herd examination a follow-up was conducted. Performance characteristics showed a clear decrease in the number of sows returning to oestrus (Table 3). The percentage of sows returning to oestrus after exclusive naturally mating decreased from 75.0 to 13.3%.

**Table 3:** Performance characteristics 5 months (October 2019- February 2020) after the affected boar was not used for mating in comparison with the status before

Performance characteristics	Status before ^a^	Status quo ^b^	Reference values [14]
Piglets born alive/ litter	13.8	12.6	12–14.0
Piglets born dead/ litter	1.8	1.1	≤ 0.5
Piglets weaned/ litter	11.1	9.8	11.0
Return to oestrus rate (%)	35.3	14.1	≤ 12.0
Regular return (%)^c^	28.7	5.9	
Irregular return (%)^d^	6.6	8.2	

^a^ Status before: Data of nine months from January 2019- September 2019 before the new boar was introduced on farm

^b^ Status quo: Data of five months from October 2019- February 2020 with the new boar on farm

^c^ Sows returning to oestrus 18–24 and 36–48 days after insemination

^d^ All other sows returning to oestrus without regular return

## Discussion and conclusions

The present report is the first description of *Actinobaculum suis* coinciding with epididymitis and pyogranuloma formation in the urogenital tract probably resulting in azoospermia in a boar. A close relative of to *Actinobaculum suis* named *Actinotignum schaalii*, formerly *Actinobaculum schaalii* [16], is described as cause for epididymitis and abscess formation in human medicine [1, 17, 18]. In pig medicine, the importance of *Actinobaculum suis* causing cystitis had been known for long time and detection methods for the agent are established [2]. However, examinations of boars, particularly pathological examinations, are very rarely carried out in Switzerland [19]. Therefore, it is likely that pathological conditions in boars are overlooked. The inflammatory reaction may have led to damage of the tubules and ductus causing spermatozoa to leak, leading to a foreign body reaction with pyogranulomatous changes. However, the route of transmission up to the epididymis has not been proven, but reflux of infected urine via the Vas deferens is described as the cause of epididymitis in humans [18, 20]. Although the urethra of boars is much longer, the pathogen is often detected at its end, making the transmission route to the testicles likely [8–12].

Since no indications for urinary tract infections in sows were detected, no further investigations were initiated. However the pathogen itself is not always associated with urinary tract infections [5] and transmission of the pathogen to the sows can therefore neither be proven nor be excluded. Finally, detailed and clustered analysis of reproductive performance data and clinical examination was crucial to detect the boar as causative agent for the increased return to oestrus rate. Regular monitoring of reproductive performance data should therefore be subject of future herd examinations. In this case, pyogranuloma and inflammation in the epididymis of a boar with poor fertility coincide with *Actinobaculum suis* and mixed flora, possibly resulting in increased returning to oestrus rate. Therefore, this ‘infection’ should be included in the differentials in boars with poor fertility. Finally, further research is needed to ensure a possible effect of *Actinobaculum suis* on the reproductive performance of a boar.

## Data Availability

The datasets used and analysed during the current study are available from the corresponding author on reasonable request.

## References

[1] Lawson PA, Falsen E, Akervall E, Vandamme P, Collins MD (1997). Characterization of Some Actinomyces-Like Isolates from Human Clinical Specimens: Reclassification of Actinomyces suis (Soltys and Spratling) as Actinobaculum suis comb. nov. and Description of Actinobaculum schaalii sp. nov. Int J Syst Bacteriol.

[2] Wegienek J, Reddy CA (1982). Taxonomic Study of ‘Corynebacterium suis” Soltys and Spratling: Proposal of Eubacterium suis (nom. rev.) comb. nov. Int J Syst Bacteriol.

[3] Ludwig W, Kirchhof G, Weizenegger M, Weiss N (1992). Phylogenetic evidence for the transfer of Eubacterium suis to the genus Actinomyces as Actinomyces suis comb. nov. Int J Syst Bacteriol.

[4] Busch M, Thoma R, Schiller I, Corboz L, Pospischil A. Occurrence of chlamydiae in the genital tracts of sows at slaughter and their possible significance for reproductive failure. J Vet Med Ser B. 2000;47(6):471–80. 10.1046/j.1439-0450.2000.00415.x. 10.1046/j.1439-0450.2000.00415.x 11014069

[5] Alberton GC, Werner PR, Sobestiansky J, Costa OD, Barioni Júnior W. Prevalence of urinary tract infections and of Actinomyces suis in urine from pregnant sows. Correlation with some physical and chemical parameters of the urine. Archives of Veterinary Science. 2000 [cited 2020 Apr 24]. p. 81–8. Available from: https://www.cabdirect.org/cabdirect/abstract/20013113896.

[6] Woldemeskel M, Drommer W, Wendt M (2002). Microscopic and ultrastructural lesions of the ureter and renal pelvis in sows with regard to Actinobaculum suis infection. J Vet Med Ser A Physiol Pathol Clin Med.

[7] Maes D, Nauwynck H, Rijsselaere T, Mateusen B, Vyt P, de Kruif A (2008). Diseases in swine transmitted by artificial insemination: An overview. Theriogenology..

[8] Pijoan C, Lastra A, Leman A. Isolation of Corynebacterium suis from the prepuce of boars. J Am Vet Med Assoc. 1983 Aug 15 [cited 2020 Apr 24];183(4):428–9. Available from: http://www.ncbi.nlm.nih.gov/pubmed/6618967. 6618967

[9] Pleschakowa V, Leibold W, Amtsberg G, Konine D, Wendt M. [The prevalence of Actinobaculum suis in boars of breeding herds in the Omsk region (Russian Federation) by indirect immunofluorescence technique]. Dtsch Tierarztl Wochenschr. 2004 Feb [cited 2020 Apr 23];111(2):67–9. Available from: http://www.ncbi.nlm.nih.gov/pubmed/15032264. 15032264

[10] Amigo CR. Development of polymerase chain reaction for Actinobaculum suis detection and phenotypic and genotypic characterization of isolates. [São Paulo]: Biblioteca Digital de Teses e Dissertações da Universidade de São Paulo; 2012 [cited 2020 Apr 24]. Available from: http://www.teses.usp.br/teses/disponiveis/10/10134/tde-21052013-151439/.

[11] Jones JET, Dagnall GJR. The Carriage of Corynebacterium suis in Male Pigs. Vol. 93, Source: The Journal of Hygiene. 1984 [cited 2020 Apr 24]. Available from: https://www.jstor.org/stable/3862825. 10.1017/s0022172400064949 PMC2129427 6501882

[12] Sobestiansky J, Wendt M, Perestrelo R, Ambrogi A. Studies on the prevalence of Eubacterium suis in boars on farms in Brazil, Portugal and Argentina by indirect immunofluorescence technique. - PubMed - NCBI. Dtsch Tierarztl Wochenschr. 1993 [cited 2020 Apr 24];100(12):463–4. Available from: https://www.ncbi.nlm.nih.gov/pubmed/8306860. 8306860

[13] Wendt M, Sobestiansky J, Bollwahn W. The treatment of Eubacterium suis infections in boars. Berl Munch Tierarztl Wochenschr. 1993 Jul [cited 2020 Apr 24];106(7):221–4. Available from: http://www.ncbi.nlm.nih.gov/pubmed/8368995. 8368995

[14] grosse Beilage E. Diagnostik und Gesundheitsmanagement im Schweinebestand Band 1. Ulmer; 2013. 21 p.

[15] Holzmann A, Busch W. Veterinärmedizinische Andrologie: Physiologie und Pathologie der Fortpflanzung bei männlichen Tieren. 2001.

[16] Yassin AF, Sprö C, Pukall R, Sylvester M, Siering C, Schumann P, et al Dissection of the genus Actinobaculum: Reclassification of Actinobaculum schaalii Lawson et al. 1997 and Actinobaculum urinale Hall et al. 2003 as Actinotignum schaalii gen. nov., comb. nov. and Actinotignum urinale comb. nov., description of Actinotignum. Int J Syst Evol Microbiol. 2015 [cited 2020 Apr 27];65:615–24. Available from: www.bacterio.net/actinobaculum.html. 10.1099/ijs.0.069294-0 25406238

[17] Tschudin-Sutter S, Frei R, Weisser M, Goldenberger D, Widmer AF. Actinobaculum schaalii - invasive pathogen or innocent bystander? A retrospective observational study. Vol. 11, BMC Infectious Diseases. 2011 [cited 2020 May 3]. Available from: http://www.biomedcentral.com/1471-2334/11/289. 10.1186/1471-2334-11-289 PMC3252262 22029906

[18] Van Aarle S, Arents NLA, De Laet K. Actinobaculum schaalii causing epididymitis in an elderly patient. J Med Microbiol. 2013 [cited 2020 Apr 24];62:1092–3. Available from: http://jmm.sgmjournals.org. 10.1099/jmm.0.048611-0 23682167

[19] Eidgenössisches Department des Inneren. Jahresbericht PathoPig. 2019;2020:1–24.

[20] Badenoch AW. Section of Urology: Vaso-Epididymal Reflux Syndrome. In: Proc R Soc Med. 1953. p. 847–9. 10.1177/003591575304601011 PMC1918707 13112202

